# In vivo three‐dimensional kinematics in unicompartmental knee arthroplasty: A sports activity cluster analysis

**DOI:** 10.1002/jeo2.70918

**Published:** 2026-09-21

**Authors:** Kenichi Kono, Ryota Yamagami, Kohei Kawaguchi, Hiroshi Inui, Shuji Taketomi, Takaharu Yamazaki, Tetsuya Tomita, Sakae Tanaka

**Affiliations:** ^1^ Department of Orthopaedic Surgery Faculty of Medicine, The University of Tokyo Tokyo Japan; ^2^ Department of Orthopaedic Surgery Faculty of Medicine, Saitama Medical Center Saitama Japan; ^3^ Department of Informatics Faculty of Informatics, Shonan Institute of Technology Kanagawa Japan; ^4^ Department of Orthopaedic Biomaterial Science Osaka University Graduate School of Medicine Osaka Japan; ^5^ Department of Medical Science Graduate School of Medicine, Morinomiya University of Medical Sciences Osaka Japan

**Keywords:** kinematics, sports activity, unicompartmental knee arthroplasty

## Abstract

**Purpose:**

The specific kinematics relevant to sports activities after unicompartmental knee arthroplasty (UKA) remain unclear. We aimed to clarify the kinematic factors associated with sports activities after UKA.

**Methods:**

Twenty‐five knees that had undergone UKA were examined fluoroscopically during squatting (weight‐bearing [WB]) and active‐assisted knee flexion (non‐weight‐bearing [NWB]). Patients who were able to perform deep squatting were included. A two‐dimensional/three‐dimensional registration technique was used to evaluate tibiofemoral kinematics, including flexion angle, axial rotation angle, varus–valgus angle, and anteroposterior translation (APT) of the femur relative to the tibia. Patients were divided into two groups using hierarchical cluster analysis based on 1‐year postoperative sports scores from the Knee Injury and Osteoarthritis Outcome Score and the 2011 Knee Society Score (low‐to‐moderate score group [*n* = 15] and high score group [*n* = 10]).

**Results:**

The sports subscale Knee Injury and Osteoarthritis Outcome Scores, except for jumping, were significantly higher in the high‐score group than in the low‐score group. During WB, the flexion angle was significantly greater in the high‐score group (136.2 ± 8.5°) than in the low‐score group (126.3 ± 14.2°). In contrast, no significant difference in flexion angle was observed during NWB. Regarding lateral APT, the high‐score group showed a more posterior position than the low‐score group throughout the flexion range during WB. No significant between‐group differences were observed in terms of axial rotation, varus–valgus angle, or medial APT.

**Conclusions:**

The ability to achieve greater flexion during weight‐bearing activities is an important factor for sports performance after UKA.

**Level of Evidence:**

Level III.

**Abbreviations**
ADL: activities of daily living
APT: anteroposterior translation
FB‐UKA: fixed‐bearing unicompartmental knee arthroplasty
KOOS: knee Injury and Osteoarthritis Outcome Score
MB‐UKA: mobile‐bearing unicompartmental knee arthroplasty
NWB: non‐weight‐bearing
PCL: posterior cruciate ligament
PROMs: patient‐reported outcome measures
TKA: total knee arthroplasty
UKA: unicompartmental knee arthroplasty
WB: weight‐bearing

## INTRODUCTION

Unicompartmental knee arthroplasty (UKA) has been shown to have superior clinical outcomes and more physiological kinematics compared to total knee arthroplasty (TKA) [1, 2, 8, 18, 25]. Moreover, return to moderate‐ or high‐impact sports after UKA is possible, with sports return rates ranging from 74% to 100% and sports return durations averaging 3–6 months [21]. Returning to lower‐impact activities is common; however, this is often because of a surgeon's recommendation or decreased motivation [21]. One study reported that patients undergoing UKA could return to high‐impact sports such as jogging, alpine skiing, judo, and singles tennis without compromising implant longevity [22]. In other words, returning to lower‐impact activities after UKA is common, but returning to high‐impact sports is also possible and safe. However, the kinematics essential for engaging in sports activities after UKA remain unclear.

Regarding activities of daily living (ADL), high knee flexion activities such as squatting, gardening, and leg strengthening exercises are correlated with patient satisfaction [26]. In addition, the kinematic difference of weight‐bearing (WB) conditions during high knee flexion activities affects the clinical outcome [4]. Moreover, the WB status during high knee flexion activities affects the UKA kinematics [9]. However, whether various high knee flexion activities show different kinematics depending on postoperative sports activity level remains unknown. Therefore, evaluation of high knee flexion activities is essential. In this study, we aimed to identify the kinematics important for engaging in sports activities after UKA. We hypothesised that patients who underwent UKA and who were engaged in sports activities would show more physiological kinematics.

## METHODS

We examined 25 knees with severe medial osteoarthritis in patients who underwent mobile‐bearing UKA (MB‐UKA). Per‐protocol analysis was used, and a total of 135 patients were excluded due to incapability of performing deep squat, osteonecrosis, and incomplete patient‐reported outcome measures (PROMs) score writing. The higher‐functioning patients who could safely perform a deep squat after surgery were examined. Each patient was asked to perform a deep WB squat and active‐assisted non‐weight‐bearing (NWB) knee flexion at a natural pace under single fluoroscopy surveillance in the sagittal plane [11]. Both activities were performed from full extension to maximum flexion. The patients practiced each motion several times prior to recording. The motions were then recorded as sequential digital radiographic images (1024 × 1024 × 12 bits/pixel, 7.5‐Hz serial spot images in a DICOM file) using a 17‐inch flat panel detector system. All images were processed using dynamic range compression for edge enhancement. A three‐dimensional (3D)‐to‐2D registration technique was used to estimate the spatial position and orientation of the femur and tibia [27]. This technique is based on a contour‐based registration algorithm that uses single‐view fluoroscopic images and 3D computer‐aided design models. The error in estimating relative motion between the femur and tibia was ≤1° for rotation and ≤1 mm for translation [12]. 3D bone models created from preoperative computed tomography scans were used for the 2D/3D registration. The following variables were measured: knee flexion angle, axial rotation and varus–valgus alignment of the femur relative to the tibia, and anteroposterior translation (APT) of the medial sulcus and lateral epicondyle. Flexion, external rotation, and valgus alignment of the femur relative to the tibia were denoted as positive values. Positive or negative APT values were defined as anterior or posterior to the axes of the tibia, respectively. All data were expressed as the mean ± standard deviation.

The patients were divided into two groups based on 1‐year postoperative PROMs, including the Knee Injury and Osteoarthritis Outcome Score (KOOS) [17] and the 2011 Knee Society Score (KSS 2011) [24] total sports scores. The PROMs and patient characteristics are shown in Tables 1 and 2. Component positioning was measured using a radiogram according to a previous study [13]. Based on hierarchical cluster analysis [10, 11], Group 1 (GR1, *n* = 15) comprised patients with low‐to‐moderate PROM scores. Group 2 (GR2, *n* = 10) comprised patients with high PROM scores. Hierarchical cluster analysis was performed using Ward's method, with the two PROMs scores standardised before analysis and equally weighted. The kinematics (knee flexion, rotation angles, varus–valgus angle, and APT) between the two groups were evaluated. All patients provided written informed consent to participate. This study was approved by The University of Tokyo Institutional Ethics Review Board (number 10462‐(2)).

**Table 1:** Patient‐reported outcome measures.

	GR1	GR2	*p*‐value
KOOS Sports/Recreations (Total 100 points)	59.7 ± 21.9	80.0 ± 9.2	<0.01
KSS 2011 Sports activities (Total 15 points)	10.3 ± 1.6	14.6 ± 0.5	<0.01

Abbreviations: GR1, group 1; GR2, group 2; KOOS, Knee Injury and Osteoarthritis Outcome Score; KSS 2011, Knee Society Score.

**Table 2:** Patients' characteristics.

Variables	GR1	GR2	*p*‐value
Age (years)	74.5 ± 7.6	71.7 ± 8.2	0.41
BMI (kg/m^2^)	24.1 ± 2.3	26.0 ± 2.7	0.13
Postoperative fluoroscopic analysis (months)	11.9 ± 4.6	10.6 ± 2.9	0.82
Sex (men: women)	4: 11	4: 6	0.58
Preoperative HKA angle (degrees)	172.4 ± 2.4	171.3 ± 5.0	0.96
Postoperative HKA angle (degrees)	175.5 ± 3.2	176.4 ± 3.7	0.60
α angle (degrees)	97.2 ± 3.6	97.3 ± 3.5	0.82
β angle (degrees)	85.7 ± 2.9	84.6 ± 1.6	0.22
γ angle (degrees)	9.3 ± 3.4	9.2 ± 4.4	0.74
δ angle (degrees)	85.6 ± 1.9	85.0 ± 2.4	0.27

*Note*: Alpha (α) angle was defined as the angle between the femoral osseous axis and the distal installation line of the femoral component on the anteroposterior view. Beta (β) angle was defined as the angle between the tibial osseous axis and the installation line of the tibial component on the AP view. Gamma (γ) angle was defined as the angle between the femoral osseous axis and the larger peg of the femoral component on the lateral view. Delta (δ) angle was defined as the angle between the tibial osseous axis and the installation line of the tibial component on the lateral view [13].

Abbreviations: BMI, body mass index; GR1, group 1; GR2, group 2; HKA, hip‐knee‐ankle.

### Statistical analyses

Commercial software (SPSS version 29 [IBM Corp.]) was used to analyse the data. Two‐way analysis of variance with Bonferroni post hoc tests was used to compare the rotation and varus–valgus angles between GR1 and GR2. A Mann−Whitney test was used to compare the PROMs and patient characteristics between the two groups. Statistical significance was set at *p* < 0.05. A power analysis was performed with Easy R (EZR) [5] software using an α error of 0.05, a (1 – β) error of 0.80 (the type II error rate should be ≤20%), and an effect size of 0.40 to compare the means between the two groups. A sample of ten knees was deemed sufficient to detect differences between GR1 and GR2.

## RESULTS

**Figure 1:**
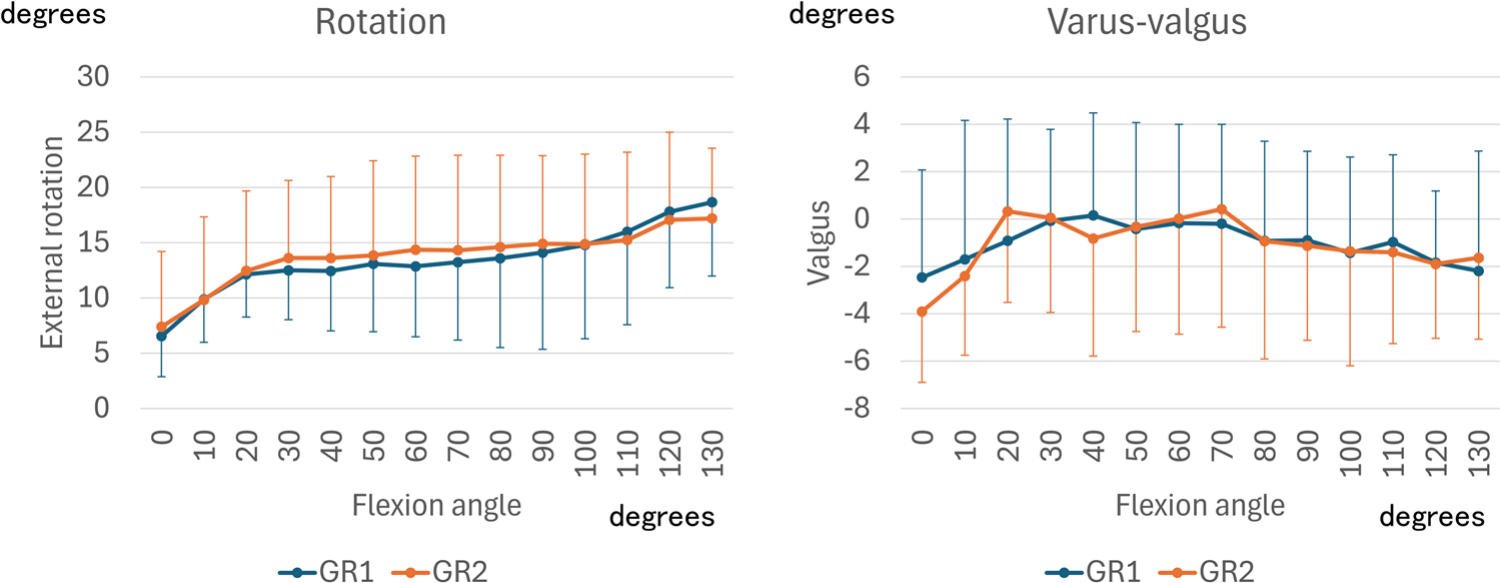
Comparison of tibiofemoral kinematics between GR1 and GR2 during weight‐bearing squatting. External rotation (left) and varus–valgus angle (right) are shown as a function of the knee flexion angle. Data are presented as mean ± standard deviation. GR1, group 1; GR2, group 2.

**Figure 2:**
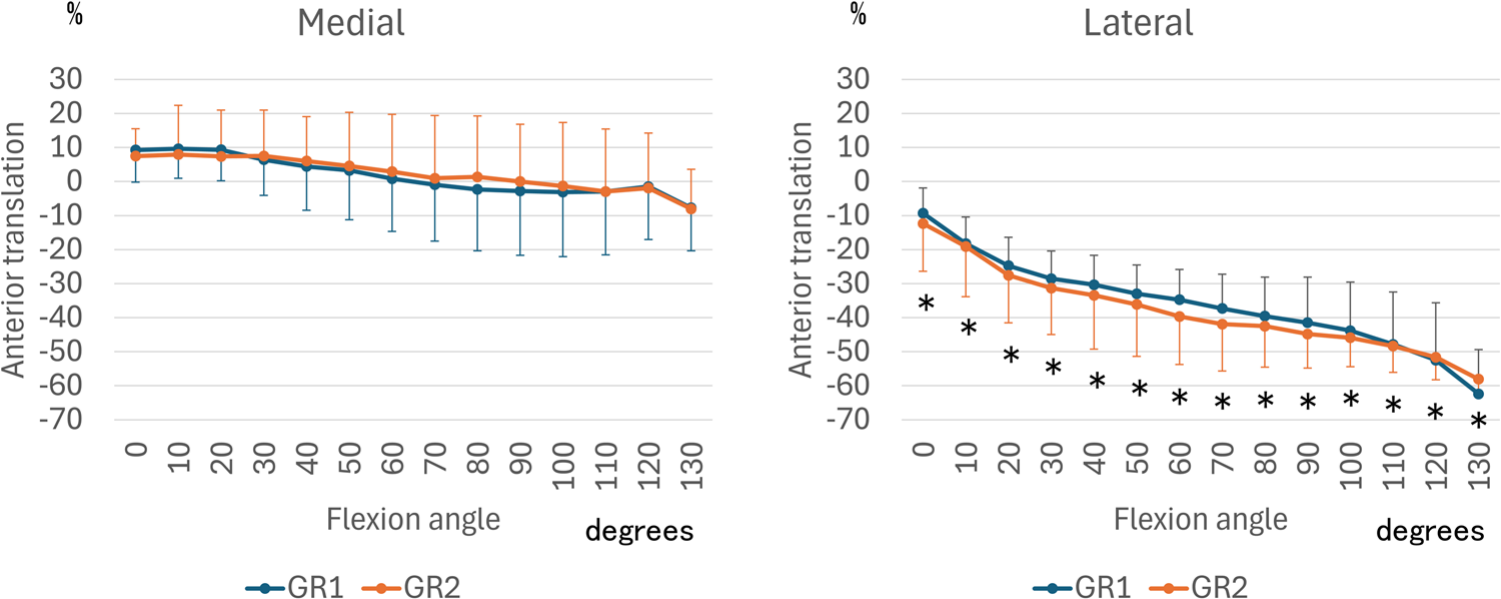
Comparison of tibiofemoral anteroposterior translation during weight‐bearing squatting between GR1 and GR2. Medial (left) and lateral (right) femoral translations relative to the tibia are shown across the flexion range. Negative values indicate posterior translation. Data are presented as mean ± standard deviation. *Significant differences between GR1 and GR2 (*p* < 0.05). GR1, group 1; GR2, group 2.

Squatting, running, and kneeling of KOOS sports subscales in GR1 were lower than those in GR2 (Table 3). In terms of kinematic analysis, GR2 exhibited a greater flexion angle during WB (GR1: 126.3 ± 14.2°, GR2: 136.2 ± 8.5°) (Table 4). The knee externally rotated with flexion (GR1: 9.0 ± 7.1°, GR2: 10.7 ± 6.0°), and no significant coronal‐plane movement was observed during WB. The rotation and varus–valgus angles were similar between the two groups (Figure 1). Concerning medial and lateral APT, both groups moved posteriorly with flexion during WB (medial; GR1: 19.1 ± 13.6%, GR2: 12.1 ± 12.3%, lateral; GR1: 43.5 ± 17.5%, GR2: 44.2 ± 17.5%). GR2 exhibited a more posterior location than GR1 in terms of lateral APT (Figure 2).

**Table 3:** KOOS (Knee Injury and Osteoarthritis Outcome Score) sports subscales.

Subscales (each 20 points)	GR1	GR2	*p*‐value
Squatting	12.7 ± 4.0	16.5 ± 2.3	<0.01
Running	11.0 ± 6.1	16.0 ± 2.3	0.02
Jumping	11.0 ± 5.2	15.0 ± 4.5	0.06
Pivoting	14.0 ± 4.9	17.0 ± 2.4	0.06
Kneeling	11.0 ± 4.9	15.5 ± 2.7	<0.01

Abbreviations: GR1, group 1; GR2, group 2.

**Table 4:** Flexion/extension angle.

	GR1	GR2	*p*‐value
WB			
Flexion angle (degrees)	126.3 ± 14.2	136.2 ± 8.5	0.047
Extension angle (degrees)	0.2 ± 3.5	2.0 ± 4.5	0.32
NWB			
Flexion angle (degrees)	129.1 ± 8.8	131.7 ± 4.7	0.36
Extension angle (degrees)	0.6 ± 2.6	1.5 ± 6.7	0.71

Abbreviations: GR1, group 1; GR2, group 2; NWB, non‐weight‐bearing; WB, weight‐bearing.

**Figure 3:**
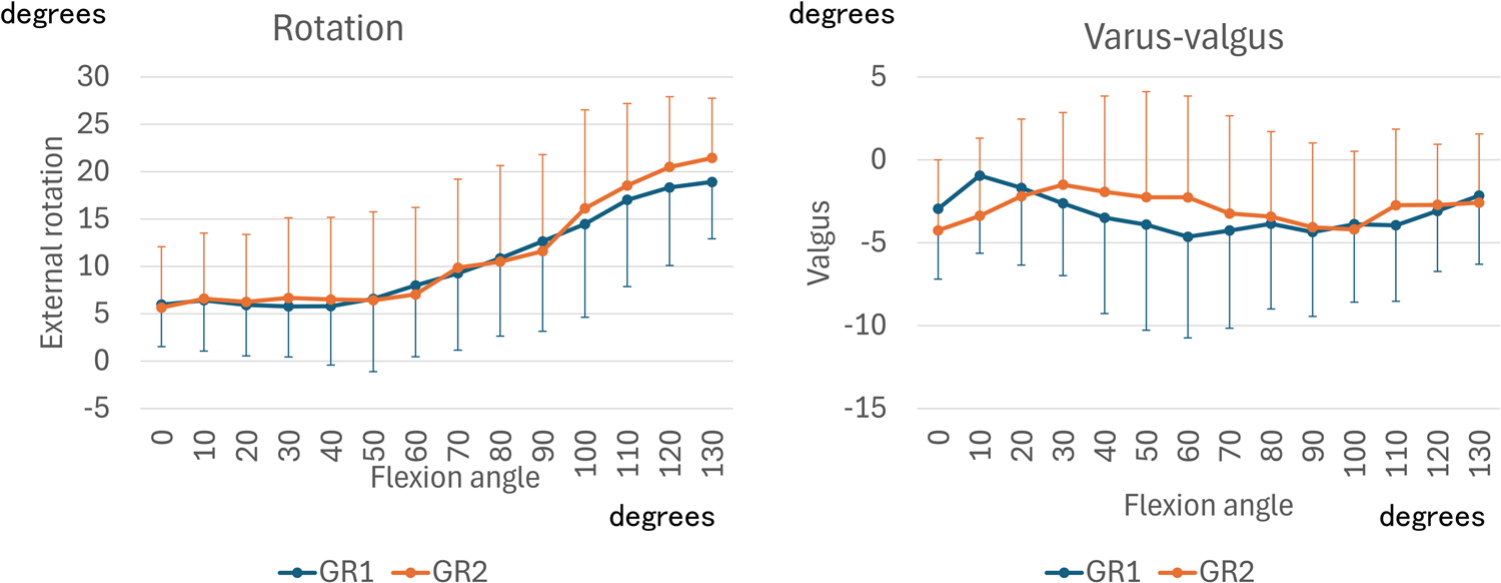
Comparison of tibiofemoral kinematics between GR1 and GR2 during non‐weight‐bearing knee flexion. External rotation (left) and varus–valgus angle (right) are shown as a function of knee flexion angle. Data are presented as mean ± standard deviation. GR1, group 1; GR2, group 2.

**Figure 4:**
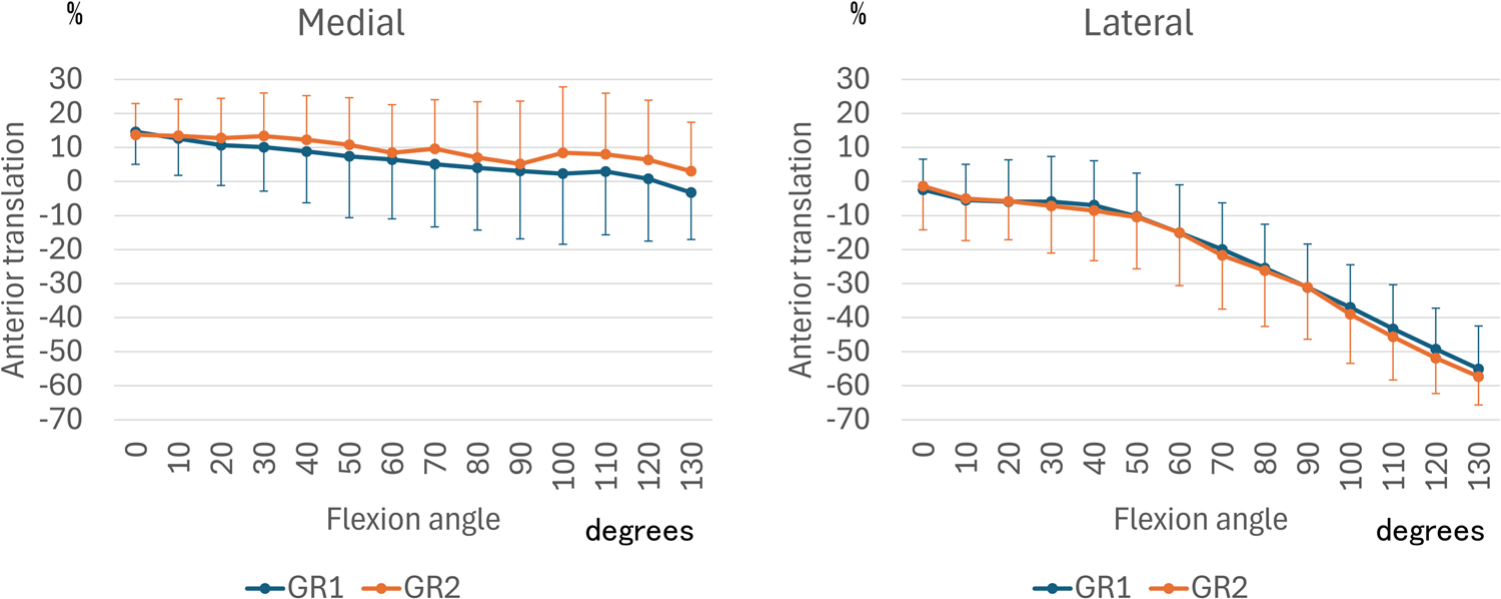
Comparison of tibiofemoral anteroposterior translation during non‐weight‐bearing knee flexion between GR1 and GR2. Medial (left) and lateral (right) femoral translations relative to the tibia are shown across the flexion range. Negative values indicate posterior translation. Data are presented as mean ± standard deviation. GR1, group 1; GR2, group 2.

During NWB, the knee externally rotated beyond 50° of flexion (GR1: 11.7 ± 5.9°, GR2: 15.0 ± 8.2°) (Figure 3). However, no significant movements were observed in the varus–valgus angle or in medial APT (Figures 3 and 4). Regarding lateral APT, both groups moved posteriorly beyond 50° of flexion (GR1: 44.1 ± 10.1%, GR2: 46.9 ± 14.2%) (Figure 4). No significant differences were observed between the two groups during NWB.

## DISCUSSION

In TKA knees, WB flexion angle and angular difference (NWB flexion angle–WB flexion angle) correlate with functional activities, ADL, and sports [4]. Therefore, it is essential to be able to squat sufficiently even when engaging in sports activities after undergoing UKA.

In addition, GR2 exhibited a more posterior location than GR1 in lateral APT during WB. In sports activities after TKA, lateral rollback during squatting motion is crucial [7]. Similarly, lateral femoral rollback during squatting appears to be important in UKA.

Lateral rollback is known to decrease in severe knee OA due to posterolateral stiffness [3, 6]. Therefore, it is important to adhere to the relevant indications for surgery when engaging in sports activities after UKA. In addition, achieving a steep tibial slope may influence the rollback, although there was no significant difference between the two groups.

It is believed that the posterior cruciate ligament (PCL) contributes to posterior stability in the flexed position during posterior‐cruciate retaining TKA [20]. Furthermore, it has been reported that PCL tension increases during the squat movement in UKA compared to healthy knees [21]. Therefore, it is possible that the increased PCL tension led to hyperstability, which contributed to the reduction in rollback.

In the GR2, the sports subscales KOOSs were also high. Moutzouri et al. reported that TKA patients with higher total KOOS Sport/Rec scores tend to experience less difficulty with each of the following movements: squatting, running, jumping, pivoting, and kneeling [16]. Furthermore, Paradowski et al. reported that the Cronbach's *α* for the KOOS Sports/Rec scale indicates very high internal consistency; additionally, all five items comprising the Sports/Rec scale load highly on the same factor in TKA [19]. Moreover, Plancher et al. reported that KOOS Sport has good person reliability and item reliability in UKA [23]. These facts suggest that to fully engage in sports activities after UKA, as after TKA, it may be necessary to be able to perform a wide range of movements—such as squatting, running, and kneeling—with equal ease.

This study has some limitations. First, we only analysed high knee flexion activities. While other ADLs, such as walking or stair‐climbing activities may display different kinematic changes, we only analysed MB‐UKA. Second, fixed‐bearing UKA (FB‐UKA) may display different kinematic changes. In other words, in terms of generalisability, this study may be limited. While previous studies have shown that MB‐UKA kinematics more closely resemble normal knee kinematics than FB‐UKA [14, 20]. Furthermore, previous finite element analysis determined that changes in these parameters in FB‐UKA appeared to induce more significant variations in the polyethylene insert, whereas in the MB‐UKA, these changes influenced bone stress distribution to a greater extent [15]. Therefore, for younger patients with relatively normal knee kinematics and high bone mineral density, considering a return to sport activities, MB‐UKA may be more suitable. Third, the sample size was relatively small. In addition, a sensitivity analysis was absent due to the difficulty of making a control because of X‐ray exposure. Therefore, this study may still be underpowered to detect smaller differences in secondary kinematic variables such as axial rotation or coronal alignment. Fourth, preoperative sports activity levels were absent because we could not evaluate the preoperative knee biomechanics due to the patients’ severe knee pain. In addition, only higher‐functioning patients were included. Therefore, it may be difficult to determine whether postoperative function reflects surgical biomechanics or baseline patient characteristics.

In conclusion, in postoperative sports activities after UKA, the ability to achieve sufficient WB flexion is crucial. Furthermore, in UKA, lateral rollback during the squat motion is essential.

## AUTHOR CONTRIBUTIONS

**Kenichi Kono**: Conceptualisation; methodology; investigation; data curation; writing—original draft; writing—review and editing. **Ryota Yamagami**: Data curation. **Kohei Kawaguchi**: Data curation. **Hiroshi Inui**: Data curation; supervision. **Shuji Taketomi**: Supervision. **Takaharu Yamazaki**: Methodology. **Tetsuya Tomita**: Supervision. **Sakae Tanaka**: Supervision.

## FUNDING INFORMATION

The authors have no funding to report.

## CONFLICT OF INTEREST STATEMENT

The authors declare no conflicts of interest.

## ETHICS STATEMENT

This study was approved by The University of Tokyo Institutional Ethics Review Board (number 10462‐(2)). Informed consent was obtained from all individual participants included in the study.

## Data Availability

The data that support the findings of this study are available from the corresponding author upon reasonable request.
